# A New Potential Resource in the Fight against Candida auris: the *Cinnamomum zeylanicum* Essential Oil in Synergy with Antifungal Drug

**DOI:** 10.1128/spectrum.04385-22

**Published:** 2023-03-28

**Authors:** M. Di Vito, S. Garzoli, R. Rosato, M. Mariotti, J. Gervasoni, L. Santucci, E. Ovidi, M. Cacaci, G. Lombarini, R. Torelli, A. Urbani, M. Sanguinetti, F. Bugli

**Affiliations:** a Dipartimento di Scienze Biotecnologiche di Base, Cliniche Intensivologiche e Perioperatorie, Università Cattolica del Sacro Cuore, Rome, Italy; b Dipartimento di Chimica e Tecnologie del Farmaco, Università di Roma Sapienza, Rome, Italy; c UOC Chimica, Biochimica e Biologia Molecolare Clinica, Dipartimento di Scienze Biotecnologiche di Base, Cliniche Intensivologiche e Perioperatorie, Fondazione Policlinico Universitario A. Gemelli IRCCS, Rome, Italy; d Department for Innovation in Biological, Agro-Food and Forest Systems DIBAF—University of Tuscia, Viterbo, Italy; e Dipartimento di Scienze di Laboratorio e Infettivologiche, Fondazione Policlinico Universitario A. Gemelli IRCCS, Rome, Italy; Agroscope

**Keywords:** efflux pump, synergy, *Galleria mellonella*, biofilm, ATPase activity

## Abstract

Candida auris is a multidrug-resistant fungus known to be a global public health problem. The skin-based transmission, together with the marked resistance to drugs, resulted in its rapid spread to all continents. The aim of this study was to identify an essential oil (EO) active in the fight against C. auris. A total of 15 EOs were tested against 10 clinical strains of C. auris. *Cinnamomum zeylanicum* EO (CZ-EO) was the most effective (MIC90 and MFC90 equal to 0.06% vol/vol). Three fractions obtained from CZ-EO, and the cinnamaldehyde (CIN), the major chemical compound, were tested to identify the principal compound effectives against C. auris. All CIN-containing samples showed anti-fungal activity. To study the synergy with fluconazole, CZ-EO, its active fraction (FR2), and CIN were tested in checkerboard tests. Results show that CZ-EO and FR2, but not CIN, synergize with fluconazole. Furthermore, only the copresence of CZ-EO or FR2 synergize with fluconazole at therapeutic concentrations of the drug (0.45 ± 0.32 μg/mL and 0.64 ± 0.67 μg/mL, respectively), while CIN only shows additive activity. *In vivo* studies conducted on Galleria mellonella larvae show the absence of toxicity of CZ-EO up to concentrations of 16% vol/vol, and the ability of CZ-EO to reactivate the efficacy of fluconazole when formulated at synergic concentrations. Finally, biochemical tests were made to study the mechanism of action of CZ-EO. These studies show that in the presence of both fluconazole and CZ-EO, the activity of fungal ATPases decreases and, at the same time, the amount of intracellular drug increases.

**IMPORTANCE** This study highlights how small doses of CZ-EO are able to inhibit the secretion of fluconazole and promote its accumulation in the fungal cell. In this manner, the drug is able to exert its pharmacological effects bypassing the resistance of the yeast. If further studies will confirm this synergy, it will be possible to develop new therapeutic formulations active in the fight against C. auris resistances.

## INTRODUCTION

Candida auris was first isolated in 2009 in Japan (1), from a woman's ear canal (hence the name “auris”). This emerging multidrug-resistant fungal pathogen is becoming a global threat to public health, attracting considerable attention due to its rapid and widespread emergence over the past decade (2). The reasons behind the recent emergence of this fungus remain a mystery to date.

Literature data suggest an independent expansion and clonal evolution of C. auris on the various continents. This theory seems to be the most probable, since before 2009 no traces of C. auris were found in historical isolates of the various continents. Furthermore, the analysis of the whole-genome sequencing (WGS) could not identify a common ancestral ancestor. The above argues in favor of a simultaneous evolution of five different clades of C. auris in the world (3 to 5).

Although C. auris belongs to the CTG clade (its constituent species translates the CTG codon as serine instead of leucine), it is a haploid fungal species that is more closely related to multiresistant species such as C. haemulonii and C. lusitaniae rather than diploid species and clinically common fungal pathogens C. albicans and C. tropicalis. In recent years, numerous hospital-acquired infections and outbreaks caused by C. auris have been recorded. Difficulty in its identification, drug resistance properties, evolution of virulence factors, associated high mortality rates (6), and long-term survival on surfaces and medical devices make C. auris particularly problematic in clinical settings (7). The Centers for Disease Control and Prevention (CDC) states that C. auris can be misidentified in laboratories lacking specific technology, such as MALDI-TOF mass spectrometry (8), which leads to probable mismanagement and outbreaks among already hospitalized patients (9).

The most worrying trait of C. auris is its inclination for drug resistance. Most isolated strains are resistant to at least one antifungal drug, most commonly fluconazole (10). The majority exhibit multidrug resistance, and some have been found to be resistant to all three major classes of antifungal drugs used in the clinical practice, such as azoles, amphotericin B, and echinocandins. This feature is not related to specific isolates from different geographic regions, and strains from each clade have been reported to be resistant to at least one class of antifungals (8). The European Committee on Antimicrobial Susceptibility Tests (EUCAST) and the Institute of Clinical and Laboratory Standards (CLSI) showed that C. auris isolates have remarkably similar resistance to fluconazole, but a wide range of different MICs, depending on the various strains for the other classes of antifungal drugs (11). Ergosterol is the main sterol component of fungal membranes and represents the target of azoles and polyenes. Fluconazole, which is the first-line antifungal drug in the clinic, has the ability to inhibit cellular ergosterol biosynthesis by targeting lanosterol demethylase, which is a cytochrome P450-dependent enzyme, essential for the production of ergosterol. The *ERG11* gene codes for lanosterol demethylase in *Candida* spp. Interestingly, three hot-spot mutations (Y132F, K143R, and F126L or VF125AL) in the *ERG11* gene were found in fluconazole-resistant strains of C. auris from different genetic clades (4). Fungal efflux pumps probably play a crucial role in the resistance mechanism of C. auris. These pumps are capable of transporting various components across the cell membrane. Some have the ability to pump drugs out of the cell, lowering their concentration and consequently abolishing the antimicrobial effect. Referring to the efflux pumps involved in antifungal resistance, there are two main families: the ATP binding cassette (ABC) and the “major facilitator superfamily” (MFS) of transporters. It is now known that overexpression of efflux pumps is one of the main mechanisms of resistance to azoles in the main pathogenic species of *Candida* (12, 13). In C. albicans, *CDR1* is a gene encoding an ABC efflux pump known for its role in azole resistance. Recently, a homologous gene to *CDR1* was found in C. auris. Furthermore, some studies have also shown that deletion of this gene could increase the susceptibility of resistant strains to azoles by 64 to 128 times (14, 15).

Currently, for the treatment of C. auris infections, because of frequent resistance to azoles, the CDC recommends initial therapy of infections with echinocandins. If the patient does not clinically respond to this treatment or has persistent mycosis for >5 days, it is recommended to switch to liposomal amphotericin B (5 mg/Kg per day) (8). In this scenario, discovery and development of new drugs is urgently needed to combat the increase of fungal azole resistance. Nowadays, essential oils (EOs), alone or in combination with conventional drugs, are used to fight human pathogens, including multidrug-resistant ones (16 [Bibr B17]to 19). EOs are mixtures of natural substances obtained from the distillation or pressing of aromatic plants with a known antimicrobial activity (20). There are several essential oils with known antifungal activity (21). Recently, using the agar diffusion method, some authors tested the antifungal activity of some EOs against C. auris (22, 23). In this study, the antifungal properties of 15 EOs were investigated, and the *Cinnamomum zeylanicum* EO (bark) was selected as the most effective. The *C. zeylanicum* EO (CZ-EO) was chemically characterized and thoroughly evaluated against azole-resistant clinical strains of C. auris. Furthermore, the efficacy of CZ-EO against C. auris was evaluated in combination with antifungal drugs. Specifically, the synergistic concentration with fluconazole was identified, highlighting the possible mechanism of action of CZ-EO in restoring the antifungal activity of fluconazole. Finally, both *in vivo* toxicity of CZ-EO and its synergistic efficacy were tested in the Galleria mellonella larval model.

## RESULTS

### Antimicrobial susceptibility test against C. auris.

In order to evaluate the susceptibility profile of clinical isolates of C. auris against drugs belonging to the three main classes of antifungals used in clinical practice, microbroth-dilution tests generally used for clinical diagnosis were performed. Table 1 shows the sensitivity profiles of the 10 C. auris to the common antifungals used in the clinic. All strains tested were fluconazole resistant (>4 μg/mL) and exhibited variable sensitivities to the other drugs.

**TABLE 1:** *In vitro* susceptibility values of C. auris against common anti-fungal drugs used in clinical practice^*a*^

Designation	FLC	MIC	CAS	SFC	POS	VOR	ITR	ANF	ANP-B
CA1	64	0.06	0.06	0.06	0.25	0.25	0.25	0.125	1
CA2	128	0.125	0.25	0.125	0.125	0.5	0.25	0.25	4
CA3	>256	0.016	0.125	0.125	>8	>8	>8	0.125	1
CA4	>256	0.125	0.25	>64	0.25	>8	0.5	0.125	4
CA5	>256	0.125	0.125	0.125	0.125	1	0.25	0.125	4
CA6	>256	0.06	0.125	0.125	0.125	4	0.25	0.06	2
CA7	>256	>8	>8	0.25	0.5	4	1	>8	4
CA8	>256	>8	2	0.125	0.25	2	0.25	>8	4
CA9	>256	0.5	>8	0.125	>8	>8	>16	1	4
CA10	>256	>8	2	64	0.25	1	0.25	>8	4

a CA1 to CA10, designation of the 10 clinical C. auris strains in study; anti-fungal drugs: FLC, fluconazole; MIC, micafungine; CAS, caspofungine; 5FC, 5-fluorcytosine; POS, posoconazole; VOR, voriconazole; ITR, itraconazole; ANF, anidafungine; ANP-B, anphotericine-B.

### Fractionation of the essential oils by low-pressure column chromatography.

To identify the main active compound responsible for the antifungal action of CZ-EO against C. auris strains, a fractionation of CZ-EO was done. Fractions are characterized by a chemical profile consisting of fewer components than the whole CZ-EO phytocomplex. Fractionations were carried out starting from the whole CZ-EO as described in section “Fractionation of the essential oils by low-pressure column chromatography” in Materials and Methods below. The fractionation process made it possible to obtain three distinct fractions. Specifically, 1 mL of FR-1 (10.5% of the initial volume), 6.8 mL of FR-2 (71.60% of the initial volume), and 0.16 mL of FR-3 (1.68% of the initial volume) were obtained from 9.5 mL of CZ-EO.

### Broth microdilution susceptibility test.

In order to select the EO most active in modulating the growth of C. auris, broth microdilution susceptibility tests were performed by testing the efficacy of 15 EOs against all the 10 fungal strains. Table 2 shows the minimal concentrations of each EO (expressed in % vol/vol) capable of inhibiting or killing 90% of the clinical strains of C. auris tested, MIC90 or MFC90, respectively. MIC90 is defined as the MIC of tested compound able to inhibit the growth of 90% of fungal strains, while MFC90 is the minimum fungicidal concentration that kills the 90% of fungal strains. The CZ-EO was the most active, with MIC90 of 0.06% vol/vol and with MFC90 of 0.06% vol/vol. *Coriandrum sativum* EO showed the second most effective activity with a MIC90 of 0.25% vol/vol and an MBC90 of 0.50% vol/vol. All other EOs showed MIC90 and MFC90 higher than the other two previously mentioned. Tween 80 alone was ineffective against C. auris strains tested.

**TABLE 2:** MIC90 and MFC90 values of the essential oils tested against 10 C. auris strains

Essential oil	% v/v	
	MIC90	MFC90
*Cymbopogon martinii*	1.00	1.00
*Cymbopogon citratus*	>4.00	4.00
*Elettaria cardamomum*	>4.00	>4.00
*Coriandrum sativum*	0.25	0.50
*Anethum graveolens*	>4.00	>4.00
*Helichrysum italicum*	>4.00	>4.00
*Cuminum cyminum*	>4.00	>4.00
*Mentha × piperita*	4.00	4.00
*Melaleuca alternifolia*	4.00	4.00
*Rosmarinus officinalis*	4.00	4.00
*Thymus vulgaris*	1.00	2.00
*Cinnamomun zeylanicum* (bark)	0.06	0.06
*Pelargonium graveolens*	4.00	4.00
*Cinnamomum camphora* (CT1 to 8 cineole)	>4.00	>4.00
*Lavandula angustifolia*	1.00	1.00

### GC-MS analysis of *C. zeylanicum* EO and their fractions.

In order to investigate the chemical composition of both the pure EO and the three fractions obtained by fractionation (section “Fractionation of the essential oils by low-pressure column chromatography”), the analyses were performed by gas chromatography-mass spectrometry (GC-MS).

The detected and identified components of both the CZ-OE from bark and the relative three fractions obtained as described in Materials and Methods (section “Fractionation of the essential oils by low-pressure column chromatography”) are listed in Table 3. As reported, only three chemical compounds of CZ-EO reached percentages greater than 5%. In detail, the main one was cinnamaldehyde (66.1%), followed by β-caryophyllene (5.6%) and (*E*)-cinnamyl acetate (5.5%). All other components were present in lower percentage values.

**TABLE 3:** Chemical composition of EO *Cinnamomum zeylanicum* and its fractions^*a*^

No.	Component	LRI*^b^*	LRI-L*^c^*	EO (%)	F1 (%)	F2 (%)	F3 (%)
1	α-thujene	918	923	0.1	n.d.	n.d.	n.d.
2	α-pinene	940	943	2.5	5.8	n.d.	n.d.
3	camphene	948	946	0.2	n.d.	n.d.	—
4	β-pinene	990	986	0.2	n.d.	n.d.	n.d.
5	α-phellandrene	998	996	0.4	n.d.	n.d.	n.d.
6	*p*-cymene	1030	1026	3.6	10.4	n.d.	n.d.
7	1,8-cineole	1031	1027	4.4	11.8	n.d.	n.d.
8	γ-terpinene	1058	1054	Tr	n.d.	n.d.	n.d.
9	cis-linalool oxide	1063	1058	Tr	n.d.	n.d.	n.d.
10	linalool	1098	1092	4.9	n.d.	n.d.	4.9
11	camphor	1131	1126	0.1	n.d.	n.d.	n.d.
12	α-terpineol	1190	1183	0.1	n.d.	n.d.	n.d.
13	*p*-anisaldheyde	1233	1229	0.1	n.d.	n.d.	n.d.
14	4-terpinenyl acetate	1291	1286	0.1	n.d.	n.d.	n.d.
15	(*E*)-cinnamaldheyde	1280	1275	66.1	n.d.	85.7	n.d.
16	eugenol	1334	1331	4.1	n.d.	n.d.	80.1
17	β-caryophyllene	1430	1426	5.6	53.7	n.d.	n.d.
18	(*E*)-cinnamyl acetate	1442	1439	5.5	8.6	7.7	6.8
19	humulene	1460	1454	0.4	4.3	n.d.	8.2
20	eugenol acetate	1488	1483	0.1	n.d.	n.d.	n.d.
21	*O*-methoxycinnamaldehyde	1508	1505	0.3	n.d.	6.5	n.d.
22	δ-cadinene	1535	1530	0.1	n.d.	n.d.	n.d.
23	caryophyllene oxide	1590	1583	0.3	5.3	n.d.	n.d.
24	benzyl benzoate	1742	1739	0.2	n.d.	n.d.	n.d.
	Sum			99.4	99.9	99.9	99.2

a The components are reported according to their elution order on apolar column. EO (%), percentage mean values of *Cinnamomum zeylanicum* EO components; F1, percentage values of fraction 1 EO components; F2, percentage values of fraction 2 EO components; F3, percentage values of fraction 3 EO components; n.d., not detected.

b Linear retention indices measured on apolar column.

c Linear retention indices from literature.

The main component of each EO’s fraction was β-caryophyllene (53.7%) in FR-1, cinnamaldehyde (85.7%) in FR-2, and eugenol (80.1%) in FR-3.

### Antifungal activity of CZ-EO fractions.

To evaluate whether and which fraction maintained the antifungal activity after fractionation, each fraction was used to perform antimicrobial tests against clinical isolates of C. auris. Table 4 shows the MIC90 and MFC90 values obtained by broth microdilution susceptibility test. As shown, FR-2 was the only fraction that showed antifungal activity equal to that of CZ-EO and cinnamaldehyde (CIN) (MIC90_FR2_ = 0.06% and MFC90_FR2_ = 0.06%). This means that cinnamaldehyde, the main component of CZ-EO and FR2, is the active compound responsible for the antimicrobial properties. The other fractions showed MIC90 and MFC90 values of >0.25% vol/vol.

**TABLE 4:** MIC90 and MFC90 values of the three fractions compared with those of CZ-EO and CIN against 10 C. auris strains

Sample^*a*^	% v/v	
	MIC90	MFC90
FR-1	>0.25	>0.25
FR-2	0.06	0.06
FR-3	>0.25	>0.25
CZ-EO	0.06	0.06
CIN	0.06	0.06

a FR-1, FR-2, FR-3, fractions of CZ-EO; CZ-EO, *Cinnamomun zeylanicum* essential oil; CIN, cinnamaldehyde.

### *In vivo* toxicity CZ-EO on Galleria mellonella.

To investigate the toxicity of CZ-EO, *in vivo* experiments conducted in the model of Galleria mellonella larvae were done. This model was chosen because it was already used to evaluate the toxicity of essential oils in general and of CZ-EO in particular (20, 24). The toxicity of the EO was evaluated by testing serial dilutions ranging from 32% vol/vol to 0.5% vol/vol on G. mellonella larvae. Data obtained (Table 5) indicate that after 24 h, the toxic dose occurred at a concentration of 32% vol/vol (100% of mortality), while for concentrations ≤16% vol/vol, no death was observed. Furthermore, only at a concentration of 16% vol/vol was a slight toxicity identified with a partial loss of motor activity, and only in some larvae, a slight pigmentation was observed.

**TABLE 5:** Toxicity of *C. zeylanicum* essential oil on G. mellonella larvae after 24 h from treatment

Essential oil (% v/v)	%	
	Alive	Dead
32	0	100
16	100	0
8	100	0
4	100	0
2	100	0
1	100	0
0.5	100	0
CTR NT	100	0

### Checkerboard titration method.

Checkerboard tests were performed to study whether the interaction between CZ-EO and fluconazole could be synergistic or antagonistic, or if the two compounds are mutually indifferent. Table 6 shows data obtained by the checkerboard titration method. Due to the strains’ resistances against the fluconazole that prevented the MIC identification of drug alone, it was not possible to calculate the fractional inhibitory concentration index (FICI). Therefore, it was necessary to consider only the FIC values of CZ-EO, FR2, or CIN to deduce its behavior when combined with the drug. The FIC values of CZ-EO and FR2 showed a synergy with the fluconazole (FIC_CZ-EO_ = 0.26 ± 0.14 and FIC_FR-2_ = 0.20 ± 0.12, respectively), while CIN showed additive activity (FIC_CIN_ = 0.52 ± 0.20). Interestingly, the concentration of the drug, in synergy with CZ-EO, falls within the range of concentrations considered effective in clinical practice tests for the choice of antifungal therapy. CZ-EO and Tween 80 (0.05% vol/vol) did not show any synergistic behavior.

**TABLE 6:** Checkerboard titration test between CZ-EO, FR2, or CIN and fluconazole^*a*^

		MIC		Combination		FIC		FICI
C	AF	C (% vol/vol)	AF (μg/mL)	C (% vol/vol)	AF^+^	C	AF	
CZ-EO	Fluco	0.07 ± 0.03	n.c.	0.02 ± 0.01	0.45 ± 0.32	0.26 ± 0.14	n.c.	n.c.
FR2	Fluco	0.03 ± 0.01	n.c.	0.01 ± 0.00	0.64 ± 0.67	0.20 ± 0.12	n.c.	n.c.
CIN	Fluco	0.02 ± 0.01	n.c	0.01 ± 0.00	0.13 ± 0.11	0.52 ± 0.20	n.c.	n.c.
TW80	Fluco	n.c.	n.c.	n.c.	n.c.	n.c.	n.c.	n.c.

a The table shows the average values. C, compound; CZ-EO, *Cinnamomun zeylanicum* essential oil; AF, antifungal drug; FR2, CZ-EO fraction two; CIN, cinnamaldehayde; TW80, Tween 80; n.c., not countable.

### Efficacy study on the formed biofilm.

**FIG 1:**
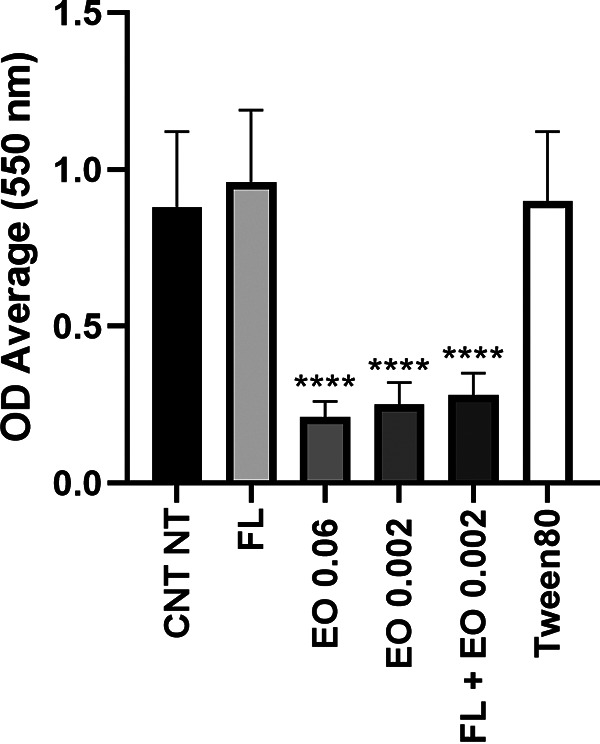
Disruptive effect on the formed biofilm. CNT NT, untreated control; FL: fluconazole 0.5 μg/mL; EO 0.06, 0.06% vol/vol of CZ-EO; EO 0.002, 0.002% vol/vol of CZ-EO; FL+EO 0.002, fluconazole +0.002 % vol/vol of CZ-EO; Tween 80, 0.05% vol/vol; ****, *P* < 0.0001.

As known, similarly to bacterial cells, *Candida* spp. also use biofilm as a form of resistance against drugs. To evaluate whether the antibiofilm capacity of CZ-EO alone or in synergistic combination with fluconazole was higher that than of fluconazole alone, appropriate microbiological tests were carried out. Tween80 alone was also tested against preformed C. auris biofilm to exclude any disrupting properties. Fig. 1 shows the demolition effect against the preformed biofilm. Tested conditions were characterized by CZ-EO at the MIC (0.06% vol/vol), and by the CZ-EO at the *in vivo* synergistic concentration (0.002% vol/vol) alone or in formulation with fluconazole. All treatments showed a statistically significant activity (*P *< 0.0001) compared with the untreated biofilm (untreated control; CNT NT) or that treated with fluconazole alone. No difference was observed comparing CNT NT with the sample treated with the fluconazole alone. These results confirm the antifungal efficacy of CZ-EO alone or in combination with fluconazole also against biofilm.

### *In vivo* efficacy study.

**FIG 2:**
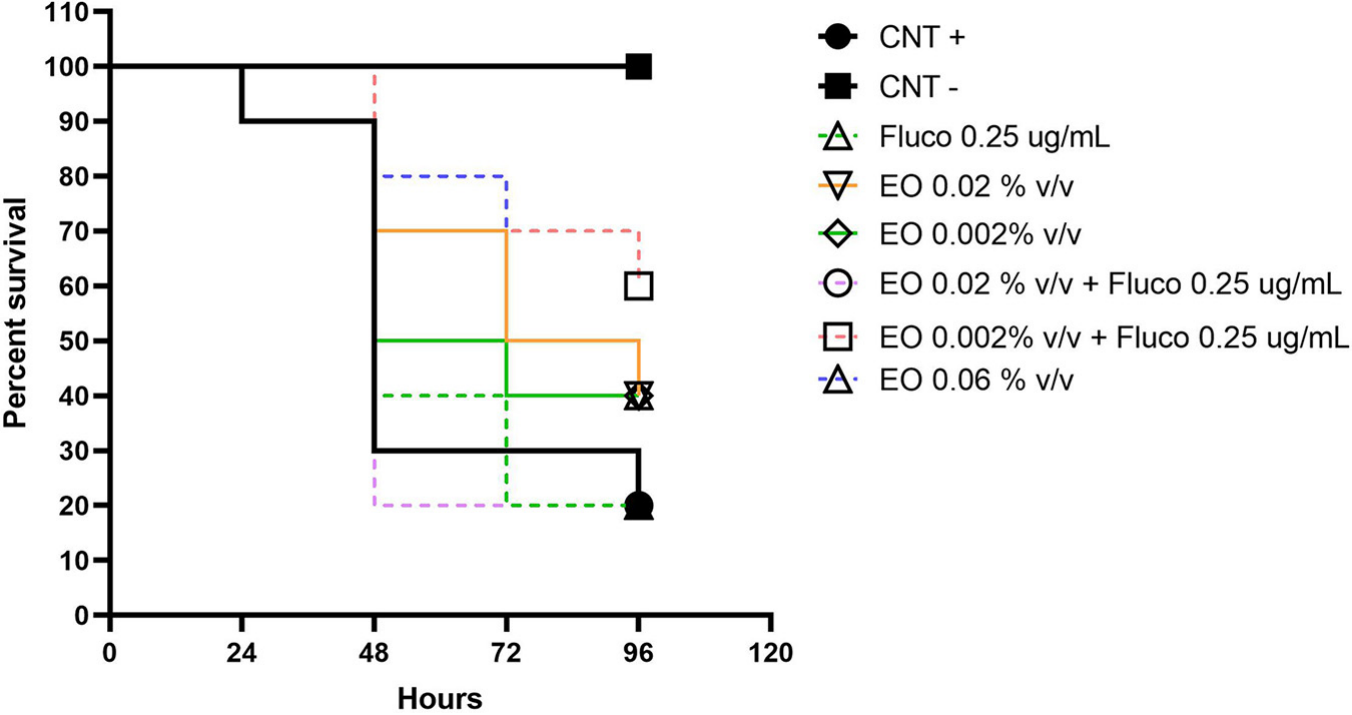
Effectiveness of treatments in G. mellonella infected with 10^7^ CFU of C. auris. CNT–, untreated group (black square/black line); CNT+, uninfected group (black circle/black line); Fluco 0.25 μg/mL, white triangle/green dashed line; EO 0.02% vol/vol, white inverted triangle/orange line; EO 0.002% vol/vol, white rhombus/green line; EO 0.02% vol/vol + Fluco 0.25 μg/mL, white circle/violet dashed line; EO 0.002% vol/vol + Fluco 0.25 μg/mL, white square/orange dashed line; EO 0.06% vol/vol, white triangle/blue dashed line.

Larvae of G. mellonella were used to investigate whether the activity of the combination of CZ-EO and fluconazole was also effective *in vivo*. Larvae infected with C. auris were treated with pure CZ-EO (at both MIC, synergistic concentration, and 10 times lower concentration than the latter). Furthermore, the combination of CZ-EO with the synergistic dose of fluconazole (0.25 μg/mL) was also investigated. Larvae were monitored for 96 h. Fig. 2 shows the *in vivo* efficacy of treatments based on CZ-EO. The survival curve of infected larvae (positive control; CNT+) was almost comparable to that of infected larvae treated with fluconazole alone (0.5 mg/mL), whereas larvae treated with CZ-EO at concentrations of 0.06% and 0.02% showed slightly significant differences at 96 h (*P*_0.06%_ = 0.041, *P*_0.02%_ = 0.047). This significance was more evident at 48 h (*P*_0.06%_ = 0.0006, *P*_0.02%_ = 0.005), while the survival of the group treated with 0.002% vol/vol of CZ-EO always remained insignificant (*P*_48h_ = 0.2, *P*_96h_ = 0.3). Specifically, the survival of the CNT+ group after 48 h was about 30%, and that of the groups treated with 0.06%, 0.02%, and 0.002% of CZ-EO was, respectively, 80%, 70%, and 50%. Finally, of the two combinations of fluconazole with CZ-EO tested, the one that after 96 h was statistically effective (*P* = 0.003) was that with the lowest concentration of CZ-EO, showing a survival of 60% compared to that of 20% for CNT+, while that with the higher concentration of CZ-EO did not show significant statistical difference compared to CNT+. In fact, the low survival of larvae treated with the higher concentration of CZ-EO + fluconazole can be explained by a toxic effect of this combination on the larva’s organism. This behavior is not observed combining fluconazole with a 10 times lower concentration of EO, which, on the other hand, is effective in counteracting yeast infectivity.

### ATPase assay in C. auris.

After evaluating the *in vivo* and *in vitro* antifungal effectiveness of CZ-EO, studies aiming to clarify the intracellular mechanism of action of EO were done. Two approaches were chosen for the study. The first aimed to confirm the inhibition of intracellular ATPase activity upon CZ-EO treatment, which has been reported in literature. The second aimed to study the ability of fluconazole to enter and remain inside fungal cells in the presence or absence of CZ-EO (see section “Quantitative determination of fluconazole” below.).

**FIG 3:**
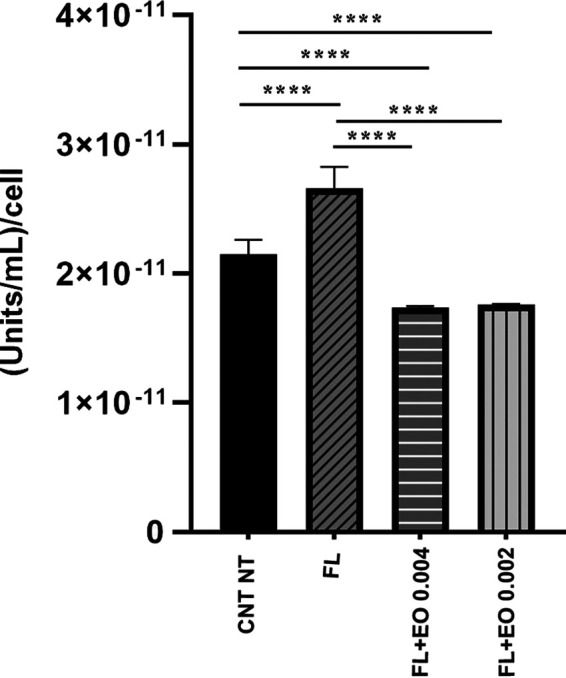
Relative ATPase activity. The enzymatic activity relative to each cell is expressed in units/mL. CNT NT, untreated control; FL, fluconazole 0.5 μg/mL; FL+EO 0.002, fluconazole + 0.004 % vol/vol of CZ-EO; FL+EO 0.002, fluconazole + 0.002 % vol/vol of CZ-EO; ****, *P* < 0.0001.

Fig. 3 shows the intracellular ATPases activity in C. auris samples treated with fluconazole alone (0.5 μg/mL) or in combination with two sub-MICs of CZ-EO (0.004% vol/vol and 0.002% vol/vol). To better observe the variation in enzymatic activity, the viability of the fungal strain was preserved by testing nonsynergistic concentrations between the drug and CZ-EO. As shown, the intracellular ATPase activity of the C. auris strain treated with only fluconazole significantly increased (*P* < 0.0001) if compared to the untreated control (CNT NT). Differently, samples treated with fluconazole combined with each of the two concentrations of CZ-EO showed a statistically significant (*P* < 0.0001) decrease in ATPase activity compared to the values of both the CNT NT and the group treated with fluconazole alone.

### Quantitative determination of fluconazole.

**FIG 4:**
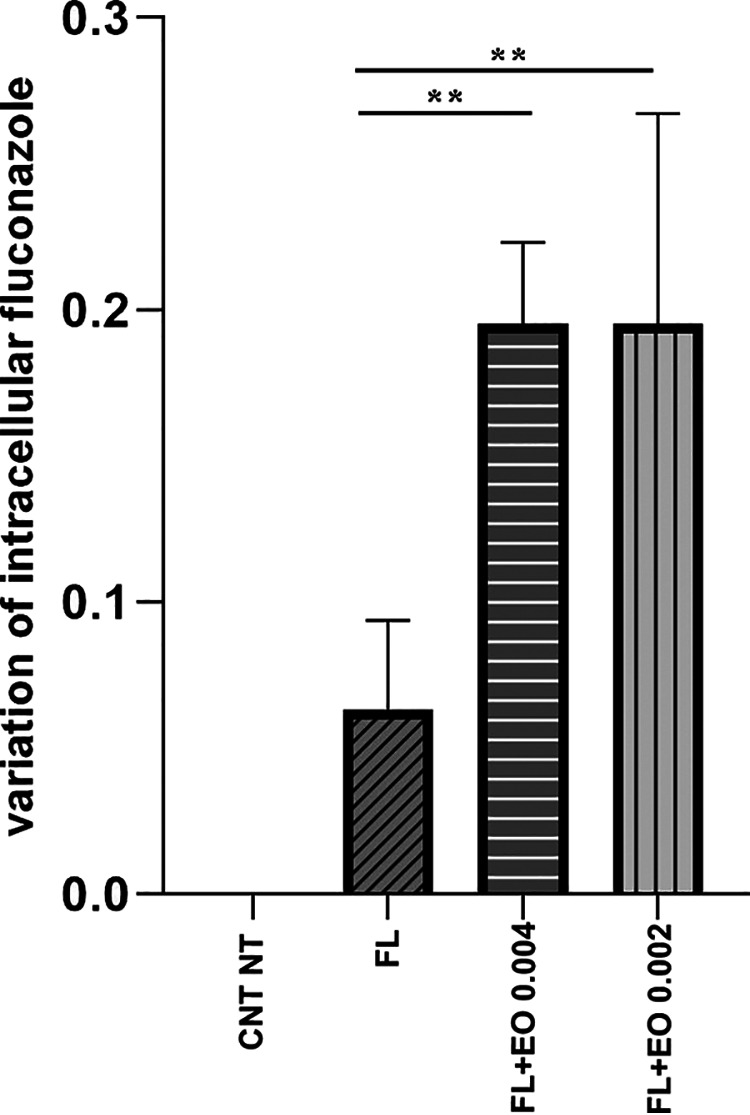
Variation relative to untreated control of intracellular fluconazole. CNT NT, untreated control; FL, fluconazole 0.5 μg/mL; FL+EO 0.002, fluconazole + 0.004 % vol/vol of CZ-EO; FL+EO 0.002, fluconazole + 0.002 % vol/vol of CZ-EO; **, *P* < 0.002.

To investigate the entry of fluconazole into the fungal cell, the method reported by Rodrigues et al. (25) was adopted and adapted. In addition to the supernatant of control characterized by the supernatant of nontreated fungal cells (CNT NT), the supernatant of cells treated with fluconazole alone and cells treated with fluconazole and CZ-EO were included as control. Fig. 4 shows the intracellular variation of fluconazole in C. auris samples treated with the drug alone or in combination with two different concentrations of CZ-EO (0.004% vol/vol and 0.002% vol/vol). The graph shows that samples treated with the combination of fluconazole and CZ-EO showed a statistically higher relative intracellular amount of fluconazole (*P *< 0.002) than that detected in the sample treated with fluconazole alone.

## DISCUSSION

C. auris has spread like wildfire in recent years, taking place in the group of nosocomial pathogens of global health interest. Initially, the spread of the strain was facilitated by the uncertain diagnosis that led to an incorrect identification of the microorganism. Subsequently, the picture was complicated by the marked resistance to numerous antifungal drugs and by the significant mortality of patients with compromised immune systems. In fact, C. auris developed a special predisposition for diffusion and transmission within and between health structures. This transmission is promoted by specific virulence factors that facilitate skin and environmental colonization (2, 5, 26). In line with what has been said, all C. auris strains considered in this study showed resistance to fluconazole and variable resistance to other antifungals (Table 1).

Recently, natural products used in traditional medicine have contributed to the development of new innovative drugs. EOs are considered complex liquid mixtures obtained from aromatic plants that comprise low molecular weight volatile substances. In order to identify the most active EOs, the preliminary study conducted on 10 clinical strains of C. auris showed that CZ-EO (bark) had the best antifungal activity (MIC90 = 0.06 and MFC90 = 0.06) followed by that of *Coriander sativum* EO (MIC90 = 0.25 and MFC90 = 0.50) (Table 2).

CZ-EO showed inhibitory capacity against some virulence factors of clinical strains of C. albicans such as the production of proteinases, the formation of germinal tubes, the interference with adhesins, and the production of hemolysins (23, 27). The antimicrobial activity of CZ-EO is expressed through different mechanisms of action: (i) alteration of the cell membrane, (ii) inhibition of ATPase, (iii) inhibition of cell division, and (iv) inhibition of the biofilm formation (27).

Literature data showed that CZ-EO is effective against *Candida* spp. strains both in liquid (24) and in vapor form (28) or in synergy with other EOs (29 [Bibr B30]to 31). *In vivo* studies showed both the effectiveness of CZ-EO in the treatment of oral *Candida* compared to nystatin, and its safety of use against removable prostheses (32, 33). Despite this, little is known about the potential of CZ-EO against infections caused by increasingly virulent nosocomial *Candida* spp., such as C. auris. To date, only one work has tested the activity of CZ-EO obtained from bark or leaf against a single strain of C. auris (NCPF 8971). This work with the methods of disc diffusion, microatmosphere, and microbroth dilution highlights the greater antifungal activity of CZ-EO obtained from bark compared to that from leaves (23).

The chemical analyses of CZ-EO used in this study are in line with what is reported in the Official European Pharmacopoeia (OEP), except for the β-caryophyllene, which was slightly more concentrated than guidelines (5.6% instead of the 1% to 4% of the OEP) (34). Three compounds reached percentage values higher than 5%: (*E*)-cinnamaldehyde (66.1%), β-caryophyllene (5.6%), and (*E*)-cinnamyl acetate (5.5%). Of these three compounds, (*E*)-Cinnamaldehyde and β-caryophyllene were entirely collected respectively in the fractions FR-1 and FR-2 obtained by the fractionation technique. On the contrary, (*E*)-cinnamyl acetate was distributed almost equally in all three fractions (Table 3). The antimicrobial test to evaluate the effectiveness of both the whole CZ-EO and its three fractions against all strains of C. auris showed that FR-2 had MIC and MFC values equal to those of the whole CZ-OE and the CIN alone (Table 4), while the other fractions, at the tested concentrations, did not show any antifungal activity. Since FR-2 was characterized by 85.7% of cinnamaldehyde and the latter is responsible for the antimicrobial activity of the EO against other *Candida* species, it can be deduced that cinnamaldehyde is also responsible for the antifungal effectiveness against C. auris.

With respect to dermal use, oral administration of CZ-EO has less restrictive indications in terms of posologies. Relating to the CZ-EO, the European Medicine Agency (EMA) assessment report indicates a daily dosage of between 50 and 200 mg administered in 2 to 3 doses (35). *In vitro* data obtained from intraperitoneal EO injections in G. mellonella did not show mortality up to the maximum CZ-EO concentration of 16% vol/vol (160 μL/mL) (Table 5). These safety data support the few data published in the literature that show an *in vivo* safety of CZ-EO greater than that obtained by *in vitro* studies of cellular models (20, 24).

From the literature, it is known that the main antimicrobial active compound of CZ-EO is the cinnamaldehyde, but little is known about its ability to synergize with antifungal drugs.

In order to evaluate the ability of CZ-EO, FR-2, and CIN to synergize with inactive antifungal drugs, checkerboard tests have been developed with fluconazole, against which all strains of C. auris were resistant. Due to the resistance of yeasts to fluconazole, it was not possible to calculate the FICI values, but the data reported in Table 6 showed that in the presence of therapeutic doses of fluconazole, lower doses of CZ-EO were necessary to obtain yeast inhibition (FIC_CZ-EO_ = 0.26 ± 0.14). Furthermore, the FIC data obtained by studying the interaction between FR-2 and fluconazole indicated that FR-2 had almost the same synergistic behavior as the EO (FIC_FR-2_ = 0.20 ± 0.12), although fluconazole, in synergy with CZ-EO, was effective in a less variable dose range than that required for FR2 (0.45 ± 0.32 μg/mL and 0.64 ± 0.67 μg/mL, respectively), whereas the study of the interaction of CIN with fluconazole shows that the CIN alone had mainly an additive behavior even if the variability showed that FIC values are borderline with the synergistic ones (FIC_CIN_ = 0.52 ± 0.20).

These data are in line with what has already been observed by Schlemmer et al. (36) regarding the synergy of CIN-fluconazole against *Malassezia pachydermatis*. In this study, no synergistic behavior of the main component of CZ-EO was highlighted. These data indicate that the synergistic activity of CZ-EO is not only due to its major chemical compound, but also to minor components, present in the phytocomplex, that are capable of synergizing with the drug. This behavior is not new in natural medicine, where complex mixtures of active ingredients included in the phytocomplex of medicinal plants have shown not only greater effects than single isolated compounds, but also less toxicity (37). The above is also valid for CZ-EO and CIN. As reported in the EMA monograph, CIN is more toxic than CZ-EO, having LD50 values equal to 2.22 g/kg and 4.16 g/kg (35), respectively. For the aforementioned reasons, our studies continued considering the activity of the whole phytocomplex instead of the main component.

Fig. 1 shows that CZ-EO alone or in synergistic concentration with fluconazole had the same demolition efficacy (*p* < 0.0001) on a preformed biofilm, whereas the fluconazole alone had no disruptive effect. Literature data show that C. auris biofilms are highly resistant to fluconazole, requiring 512 times higher azole concentration than the respective MICs (38) and ordinary disinfectants (2, 5, 26). On the contrary, the demolition activity of CZ-EO and CIN on *Candida* spp. biofilm is almost equal to the MIC values (39). Therefore, the effectiveness of CZ-EO and CIN could be a valid support in the prevention of environmental contamination by C. auris.

In order to evaluate a possible systemic use of CZ-EO, the *in vivo* efficacy data obtained in G. mellonella larvae are interesting. Data indicate that up to 48 h, both the MIC (0.06% vol/vol) and sub-MIC (0.02% vol/vol) concentrations of CZ-EO were able to inhibit the systemic infection of C. Auris in 80% and 70%, respectively, of the larva, in a statistically significant manner (Fig. 2), whereas the administration of a single dose characterized by a very low concentrations of CZ-EO (0.002% vol/vol) added to therapeutic concentrations of fluconazole (0.5 μg/mL) could preserve up to 48 h the survival of the 70%, and up to 96 h the 60% of larvae compared to the 20% observed at both 48 h and 96 h in the untreated group. These *in vivo* data indicate that it is theoretically possible to reactivate the efficacy of fluconazole in resistant strains by joining therapeutic doses of fluconazole at very small and safe daily doses of CZ-EO.

**FIG 5:**
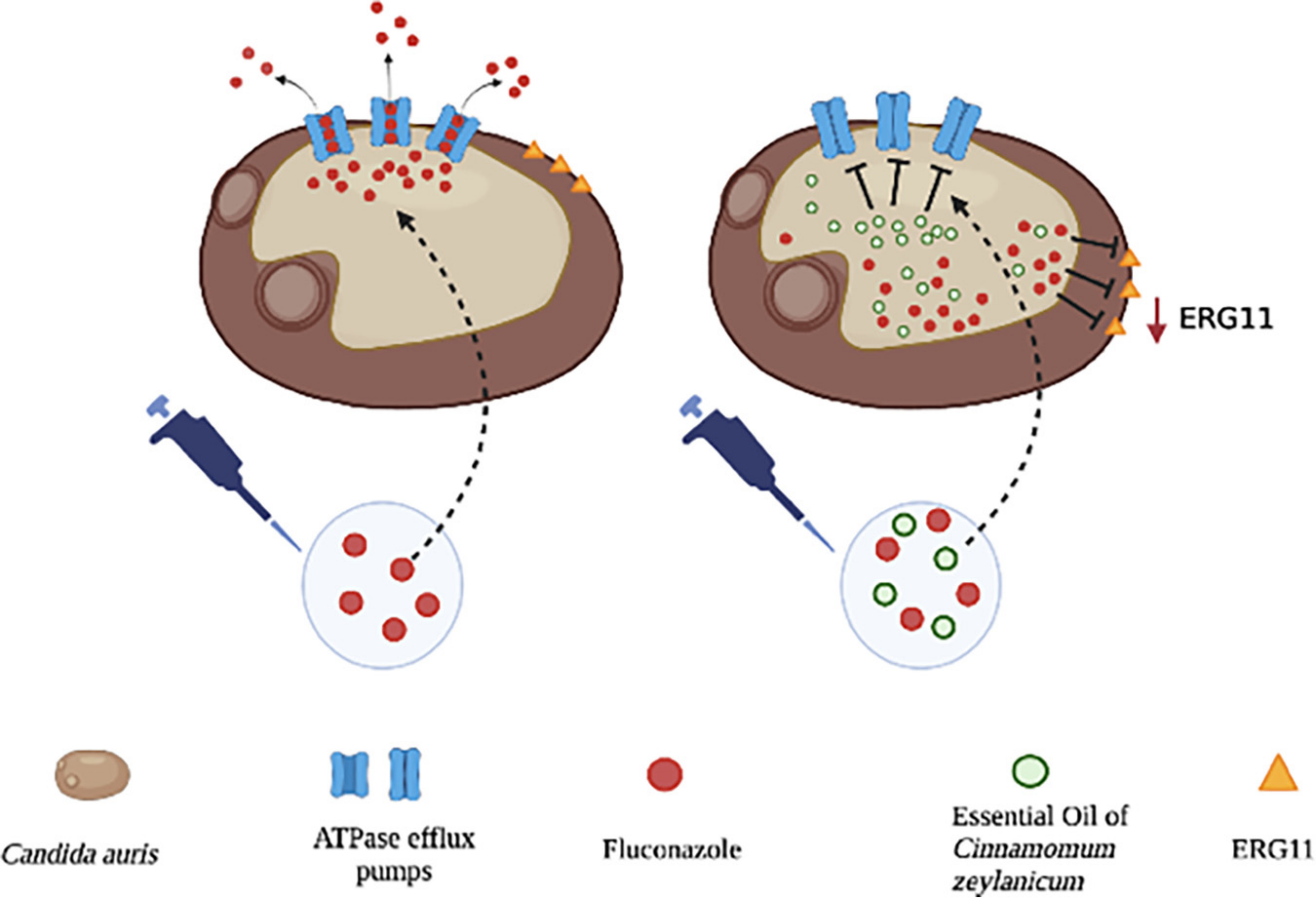
Mechanism of action of fluconazole alone or in synergy with CZ-EO. The resistance of the C. auris cell to fluconazole is shown on the left. The drug is extruded through the ATPase pumps. On the right, the efficacy of fluconazole in the presence of CZ-EO is shown. In the cell, EO inhibits the ATPase pumps, and fluconazole can carry out its antifungal activity.

Although the specific fungal mechanism of resistance to fluconazole is not yet known, it can be assumed that this involves ATP-dependent active transport pumps as known and documented for other species belonging to the genus of *Candida* (40) (Fig. 5a).

The data obtained from the study of ATPase activity (Fig. 3) support this hypothesis by showing that in the presence of fluconazole, the intracellular ATPase activity increased in a statistically significant manner (*P *< 0.0001) if compared with the untreated control (CNT NT), allowing the cell to eliminate the fluconazole externally. On the contrary, the samples treated with nonlethal associations of fluconazole and CZ-EO showed that the enzymatic activity was statistically reduced to values lower than those of the CNT NT. These last data suggest that the cell may lose its ability to eliminate the harmful compound. The hypothesis is further supported by the analysis of intracellular fluconazole (Fig. 4), which significantly increased (*P *= 0.004) in the presence of CZ-EO if compared to the sample treated with fluconazole alone.

According to what has been observed, it should be possible to conclude that in the presence of very low doses of CZ-EO, the ATPase pumps are in some way inactivated and fluconazole is blocked inside the cell, where it becomes active again, toward its target (Fig. 5b). To support this hypothesis, articles by Shreaz et al. show that antifungal effects of CIN were exerted by targeting H^+^ ATPase. Also, these authors demonstrated a decrease in proton extrusion in *Candida* spp. treated with the MIC value of CIN (41, 42).

### Conclusions.

In conclusion, the results indicate the antifungal efficacy of CZ-EO extracted from bark against C. auris strains for MIC and MFC concentrations of 0.06% vol/vol. Furthermore, CZ-EO can synergize with fluconazole. Tests aimed at defining the mechanism of action of the CZ-EO when used in synergy with fluconazole indicate that, with reasonable certainty, the EO used at low concentrations likely inhibits the activity of ATPase pumps, and consequently the ability of the cell to eliminate the drug that is harmful to it. The fluconazole, thus imprisoned in the cell, regains its ability to inhibit the synthesis of ergosterol leading the cell to death. Therefore, even if further studies are needed to confirm on a larger scale, both *in vitro* and *in vivo*, what has been observed regarding the efficacy and mechanism of CZ-EO action, CZ-EO represents a valid resource in the fight against the spread of C. auris and its resistances. If the data is confirmed, CZ-EO can be used in synergy with antifungal drugs in the *in vivo* treatment of infected critically ill patients.

## MATERIALS AND METHODS

### Clinical strains of C. auris.

Ten clinical Italian isolates of C. auris, never characterized or used in other studies, were included in the study. The strains were kept and eventually available from our institute's strain collection after personal contact. Used strains were isolated from positive blood cultures and cultured in Sensititre YeastOne Broth (Thermo Fisher, Waltham, Massachusetts, United States) at 37°C for 24 h to evaluate the MICs (MIC and MIC90) and in Sabouraud Dextrose Agar (Oxoid, Wade Road, Basingstoke, Hants, UK) to evaluate the Minimum Fungicidal Concentrations (MFC and MFC90).

### Essential oils.

The following 15 EOs were tested against the C. auris strains: Cymbopogon martinii, Cymbopogon citratus, Elettaria cardamomum, Coriandrum sativum, Anethum graveolens, Helichrysum italicum, Cuminum cyminum, Mentha piperita, Melaleuca alternifolia, Rosmarinus officinalis, Thymus vulgaris with thymol chemotype, Cinnamomum zeylanicum (bark), Pelargonium graveolens, Cinnamomum camphora, and Lavandula angustifolia (Pranarôm International, Ghislenghien, Belgium). According to the manufacturer's description, all the EOs were obtained by low-pressure steam distillation.

### Standards and materials.

Tween 80 was purchased from Fisher Chemical (Fisher Scientific, Geel, Belgium). Natural cinnamaldehyde, obtained from *Cinnamomum* spp. EO (purity > 98%) (43), was purchased from Ventos (Barcelona, Spain, batch L4412580). Broth microdilution test, checkerboard test, and biofilm cultures were developed using the Sensititre YeastOne YO10 AST Plate (Thermo Fisher, Waltham, MA, USA) and the Sabouraud agar (Oxoid, Wade Road, Basingstoke, Hants, UK) to identify MFC values. Crystal violet staining (Sigma-Aldrich, UK) was used to study the biofilm disintegration. Chemicals and solvents were of analytic or pharmaceutical grade, while petroleum ether and ethyl acetate used to obtain fractions were purchased from Sigma-Aldrich (Steinheim, Germany). PBS (Alfa Aesar, Ward Hill, Massachusetts, United States) and 24-well plates (Corning Incorporated, Kennebunk, ME, United States) were used for fungal cell culture. Glass beads (452 to 600 μm by Sigma-Aldrich, St. Louis, MO, USA) were used to study the ATPase activity measurements.

### Antimicrobial susceptibility test against C. auris.

An antimicrobial susceptibility test against C. auris clinical isolates by using anidulafungin, micafungin, caspofungin, 5FC, posoconazole, voriconazole, itraconazole, fluconazole, amphotericin B, FR59, and fenticonazole was performed according to the manufacturer's instructions. MIC values were classified as sensitive (S), susceptible to increased exposure (I), and resistant (R) as indicated by the manufacturer’s instructions, and according to the Institute for Clinical and Laboratory Standards for Antimicrobial Susceptibility Testing (CLSI).

### Broth microdilution susceptibility test.

Broth microdilution susceptibility tests were performed against all 10 fungal strains studied according to the European Committee on Antimicrobial Susceptibility Testing (EUCAST) international guidelines. The antifungal effectiveness of all EOs and the fractions of CZ-EO (obtained as described in section “Fractionation of the essential oils by low-pressure column chromatography” in Materials and Methods, below) were tested.

Briefly, a cell suspension equal to 5 × 10^5^ CFU/mL was obtained, and 50 μL of each fungal suspension was dispensed in each well of a 96-well microtiter plate. Serial dilutions ranging from 4% vol/vol (40 mL/L) to 0.007% vol/vol (0.07 mL/L) for each EO were tested except for CZ-EO and their fractions, which were tested at concentrations ranging from 1% vol/vol (10 mL/L) to 0.002% vol/vol (0.02 mL/L). To emulsify the EOs and CZ-EO fractions, 0.05% vol/vol of Tween 80 was utilized. Suitable positive controls, only with culture medium, were made for each microbiological strain. After 24 h of incubation at 37°C, MIC values were visually determined. The MIC was defined as the lowest concentration that allowed a complete inhibition of the organism’s growth compared with the growth in the substance-free control. Tween 80 was always used alone to exclude interference with cell viability. To determine the minimum fungicidal concentration (MFC), 5 μL of the content of each well was seeded on Sabouraud agar plates, which were incubated at 37°C for 24 h. The MFC was defined as the lowest concentration that killed 99.9% or more of the initial inoculum cells. All tests were performed in triplicate and on different days.

### Fractionation of the essential oils by low-pressure column chromatography.

Low-pressure column chromatography (LPLC) using silica gel 60H (0.04 to 0.063 mm) (Macherey-Nagel, Germany) as the stationary phase was used to fractionate CZ-EO. Sixty grams of silica gel were mixed with 200 mL of petroleum ether and stirred to obtain a pourable slurry that was packed into a glass column with a cotton plug and a sand layer at the bottom. After the stationary phase was accurately packed, 9.5 mL of CZ-EO was gently applied to the top of the column bed on a sand layer. The following solvents were used: petroleum ether/ethyl acetate (90/10), petroleum ether/ethyl acetate (75/25), and petroleum ether/ethyl acetate (50/50). Solvents were added sequentially (180 mL each), dropping the mobile phase under gravity. Three fractions (FR-1, FR-2, and FR-3) were manually collected, and solvent evaporation was carried out by a rotatory evaporator with vacuum (RV08-VC, IKA, USA) at room temperature (44). At the end of the fractionation, each fraction was reconstituted in 9.5 mL (equal to the initial volume of CZ-EO) of Sensititre YeastOne YO10 AST and 0.05% vol/vol of Tween80 in order to have the same concentration of active compounds present in the unfractionated CZ-EO. Fractions were stored at +4°C until microbiological tests.

### Headspace (HS) sampling.

To describe the volatile chemical composition of pure and formulated *C. zeylanicum* EO, a Perkin Elmer Headspace Turbomatrix 40 (Waltham, MA, USA) autosampler connected to GC-MS was used. About 2 mL of each sample was individually placed into a 20-mL headspace glass vial with crimp seal with PTFE/silicone septa. The procedure was as described previously, with some modifications (45).

### GC-MS analyses.

A Clarus 500 model Perkin Elmer (Waltham, MA, USA) gas chromatograph coupled with a mass spectrometer and equipped with FID (flame detector ionization) was used to describe the chemical composition of fractions and EO. The GC oven was equipped with a Varian Factor Four VF-1 capillary column, and He was used as the gas carrier at a flow rate of 1.0 mL min^−1^ in constant flow mode. The GC temperature program was as follows: initially at 60°C and then ramp up to 220°C at a rate of 6°C min^−1^ for 20 min. An electron impact ionization (EI) system was used at 70 eV in scan mode in the range of 35 to 400 *m/z*. The identification of compounds was performed by matching their mass spectra with those stored in the Wiley 2.2 and Nist 02 mass spectra libraries database and by comparison of their linear retention indices (LRIs), relative to C_8_–C_30_ n-alkanes, with those available in the literature. Relative quantifications of compounds were expressed as a percentage of the peak area to that of the total peak area without the use of an internal standard and any factor correction. All analyses were carried out in triplicate.

### Checkerboard titration method.

The checkerboard titration method was used to study the interaction between CZ-EO, or its active fraction (FR2) or CIN, and fluconazole. Specifically, the following concentration ranges were tested: from 0.25% vol/vol (2.5 mL/L) to 0.002% vol/vol (0.02 mL/L) for CZ-EO, FR-2, and CIN; from 256 μg/mL and 0.0015 μg/mL for fluconazole. Tests were performed using 96 microplates (Falcon, Corning Incorporated, New York, USA) incubated for 24 h at 37°C. A combination of scalar dilutions between CZ-EO and Tween 80 was tested to verify eventual synergism between the compounds. At the end of the incubation time, the MIC values relative to the single compounds and their synergies were measured to obtain the values of the inhibiting fractional concentrations (FICs) and the related indices of inhibiting fractional concentrations (FICIs) in accordance with the EUCAST international guidelines (46). The fractional inhibitory concentration index (FICI) was used to study the interactions between the various compounds as follows: synergy (FICI ≤ 0.5), additive effect (0.5 < FICI ≤ 1), indifference (1 < FICI ≤ 2), and antagonism (FICI ≥ 2). All tests were performed in triplicate and on different days.

### Effectiveness of CZ-EO on the formed biofilm.

A clinical high biofilm producer strain of C. auris (strain CA10) was grown overnight in RPMI broth (Sigma-Aldrich). To test for disaggregation of biofilm, cells were diluted in RPMI broth (LB, Sigma-Aldrich) to a turbidity of 0.5 McFarland. The strain was grown in 96-well plates (Thermo Fisher Scientific, MA, USA) at 37°C for 3 days, and then the biofilm was treated for a further 24 h with or without compounds. After the incubation period, biofilm was washed three times with PBS and cells fixated in acetone for 10 min. Each treatment was eight times. Tween 80 alone was used in parallel to exclude interference of the compound with biofilm desegregation or synergism with fluconazole. In addition to the untreated control (CNT NT), the following treatments were studied: 0.5 μg/mL of fluconazole, 0.002% and 0.06% vol/vol of CZ-EO, 0.5 μg/mL of fluconazole + 0.002% vol/vol of CZ-EO. The resultant biofilms were stained with crystal violet staining for 30 min. Subsequently, the stained biofilm was washed in PBS, and 100 μL/well of ethanol was added to completely dissolve the crystal violet. Finally, the absorbance at 560 nm was detected. All tests were performed eight times and on different days.

### *In vivo* safety and toxicity study on Galleria mellonella.

To evaluate the safety of CZ-EO, i*n vivo* studies on G. mellonella larvae were conducted. Larvae were individually weighed before the treatment (0.3g to 0.45g body weight). Scalar dilutions ranging from 32% vol/vol to 0.5% vol/vol were tested. Ten milliliters of each treatment were injected into the last hind leg of larvae. Each group was characterized by 10 larvae. Syringes of 0.5 mL without dead volume were used for the injections. Before the treatment, the area was disinfected with 70% ethanol. The larvae were incubated in the dark at 33°C for 24 h. At the end of the incubation, the dead larvae were counted. In order to evaluate the effectiveness of CZ-EO alone or in synergy, *in vivo* studies were conducted on G. mellonella. Specifically, the effectiveness of the following five final concentrations were tested: 0.25 μg/mL of fluconazole; 0.2% and 0.002 vol/vol of CZ-EO; 0.02% vol/vol of CZ-EO + 0.25 μg/mL of fluconazole; 0.002% vol/vol of CZ-EO + 0.25 μg/mL of fluconazole. Seven groups of 30 larvae/each infected by injecting into the last hind leg 10 μL of a suspension containing 1 × 10^7^ CFU/mL of the C. auris (strain CA10) were considered. Five of these groups were simultaneously treated with 10 μL of one of the treatments at the concentrations indicated above, and the sixth group was not treated and was considered the positive control (CNT+). The seventh group was treated by injecting only 10 μL of sterile water and considered the negative control (CNT). Subsequently, larvae were incubated in the dark at 33°C for 4 days. The survival was monitored daily, and larvae were considered dead when they did not respond to stimulation and changed color to dark. All experiments were performed at least in triplicate. All tests were repeated three times on different days.

### Cell culture to study the CZ-EO mechanism of action.

To evaluate both the ATPase activity and the cellular intake of fluconazole, the method described by Rodrigues et al. (25), with some small changes, was performed. Specifically, in Sensititre YeastOne Broth, an inoculum of the clinical strain of C. auris (strain CA10) was incubated at 37°C for 24 h in gentle agitation (120 rpm). At the end of the incubation, cells were collected and centrifuged (4,000 RPM, 10 min, 4°C), and the pellet was washed twice with PBS. Subsequently, the pellet was resuspended in broth to a final suspension of 1 × 10^5^ CFU/mL. Aliquots of 500 μL of the fungal suspension were placed in 24-well plates and incubated at 37°C for 24 h. After 24 h, 250 μL of culture medium was replaced with an equal volume of medium containing the tested concentrations, and the plates were closed with an adhesive film for micromethods and placed at 37°C for 24 h in gentle agitation (120 rpm). To avoid cell death, three treatments with sub-MICs (fluconazole 0.5 μg/mL; CZ-OE at 0.004% vol/vol + fluconazole 0.5 μg/mL; CZ-OE at 0.002% vol/vol + fluconazole 0.5 μg/mL) were studied. Untreated sample was considered control. Plates were both sonicated for 45 min and scraped to detach the adherent cells. Subsequently, the contents of wells were collected and centrifuged at 14,000 rpm for 10 min, and the super was collected and stored at −80°C until fluconazole intake analysis (see section “Fluconazole cellular uptake” below). The pellet was washed twice in PBS and used to study the ATPase activity (see the following section). All tests were performed in triplicate and on different days.

### Study of ATPase activity.

Pellets obtained from treatments described in the preceding section were resuspended in water in order to have a suspension of about 1 × 10^6^ CFU/mL. Pellets were slightly disrupted with a mechanical method by adding a volume of glass beads equal to half the volume of the fungal suspension. Subsequently, cells were centrifuged three times for 10 s in Mini-Beadbeater-16 (BioSpec Products, Inc., Bartlesville, USA). Between centrifuges, the suspension was placed on ice for 30 s. At the end of the treatment, the supernatant was used to study the ATPase activity. The ATPase Assay kit (Abcam; Cambridge, UK) was used for the study. The assay was performed according to the manufacturer's instructions. For each sample, variations of intracellular ATPase activity related to the control sample were considered. All tests were performed in triplicate and on different days.

### Fluconazole cellular uptake.

The fluconazole was measured with a Chromsystems diagnostic kit (MassTox TDM Series A) for UPLC (ultraperformance liquid chromatography)-MS/MS assay of antimycotic drugs. The UPLC-MS/MS system consisted of an UPLC and autosampler Acquity I-Class (Waters Corporation, Milford, MA, USA) and a triple quadrupole mass spectrometer Xevo-TQs Micro (Waters Corporation, Milford, MA, USA) equipped with an electrospray ion source. Analyses were performed in positive ion mode. The optimized parameters for the ion source were desolvatation temperature at 550°C, desolvatation gas flow at 1,100 L/h, cone gas flow 10 L/h, and capillary voltage at 3.0 kV. The multiple reaction monitoring (MRM) transitions for fluconazole and fluconazole internal standard, their respective collision energy, cone voltage values, and dwell time are reported in Table 7.

**TABLE 7:** Technical parameters of UPLC-MS/MS assay

Compound	Q1 mass (Da)	Q3 mass (Da)	CE^*a*^ (volts)	Cone (volts)	Dwell time (s)
Fluconazole	308.1	220.0	18	40	0.060
Fluconazole IS	311.1	223.0	18	40	0.060

a CE, collision energy.

The chromatographic separation was performed with a gradient of mobile phase A (MassTox TDM Series A) and mobile phase B (MassTox TDM Series A), with a flow rate of 0.6 mL/min. The gradient followed the following pattern: 0 to 0.5 min 30% B, 0.5 to 0.51 min 100% B, 0.51 to 2.80 min 100% B, 2.80 to 2.81 min 30% B, 2.81 to 3.20 min 30% B. The oven temperature was set at 25°C. The injection volume was 5 μL, and the total analysis time was 3.2 min. Calibration was performed using a four-point calibration curve. Calibration points were treated according to the protocol, and calibration curves were constructed by plotting peak area ratio (analyte/International Standard [IS]) versus nominal concentration. The analytical process was monitored using two-level quality controls. Samples, calibrators, and quality controls were prepared according to the manufacturer’s instructions. Briefly, 25 μL of extraction buffer was added to 50 μL of the sample, and after vigorous agitation, the sample was incubated 2 min. Then, 250 μL of a mixture containing precipitation reagent and internal standard was added, and after vigorous agitation the sample was centrifuged at 15,000 × *g* for 5 min. Twenty microliters of supernatant were diluted with 20 μL of dilution buffer transferred into the vials, and 50 μL was injected into the UPLC-MS/MS system. Data acquisition was carried out using the mass spectrometer software MassLynx v.4.1 (Waters corporation, Milford, MA, USA), while the processing software TargetLynx XS (Waters corporation, Milford, MA, USA) was used for quantitative analysis. All tests were performed three times on different days.

### Statistical analysis.

The statistical analysis was performed using the software GraphPad Prism v.8 (GraphPad Software Inc., San Diego, CA, USA). To study both the effectiveness of all treatments on the biofilm and their toxicity on G. mellonella, an ordinary Dunnett’s (*P *< 0.05) multiple-comparison test was applied. Comparisons were made between each treatment and CNT NT or CNT+, respectively, in the biofilm and in the toxicity tests. Normal distribution data were analyzed using mean and standard deviation parameters.
